# A National Dental Library and Museum

**Published:** 1896-01

**Authors:** Wms. Donnally

**Affiliations:** Washington, D. C.


### A National Dental Library and Museum.

BY WMS. DONNALLY, WASHINGTON, D. C.

Abstract of paper read at American Dental Association, August, 1895.

"There are now 150 public museums in the United Kingdom, all active and useful. While museums in their too rather vague divisions of science and art attempt to cover the whole field of human thought and interest, there is an increasing tendency to specialization, and from this we may expect a nearer approach to perfect work and a more direct influence upon the separate departments of human activity."

The writer then cited the advantages of the Army and Medical Museum and Library at Washington, as the proper place for the institution of a national museum and library. It is stated that the Medical Library, in March, 1895, contained 114,567 bound volumes and 183,778 pamphlets. The library contains three-fourths of the medical literature of the past ten years. There are about 53,000 visitors to the museum and library annually, besides the library is used by over 3,000 students annually.

After citing the advantages of this institution, he said: "Never was there such an opportunity freely offered a profession to safely and certainly perpetuate the fame and effect of its achievements, to give it a higher place among the learned callings, to acquaint the profession and the general public with its acquirements, and to secure to it a depository of all things of present or future historical and educational value—a depository already famed as the richest in medical lore and further enhanced by its unequaled index-catalogue. You are urged to consider the invitation of Dr. Billings, to formally adopt this government museum and library as your own National Dental Museum and Library."

